# Gonioscopically Guided Nonpenetrating Cyclodialysis Cleft Repair: A Novel Surgical Technique

**DOI:** 10.5005/jp-journals-10008-1218

**Published:** 2017-01-18

**Authors:** Ian AS Rodrigues, Brinda Shah, Saurabh Goyal, K Sheng Lim

**Affiliations:** 1 Specialist Registrar, Department of Ophthalmology, St Thomas’ Hospital, London United Kingdom; 2 Consultant, Department of Ophthalmology, St Thomas’ Hospital, London United Kingdom; 3 Consultant, Department of Ophthalmology, St Thomas’ Hospital, London United Kingdom; 4 Consultant, Department of Ophthalmology, St Thomas’ Hospital, London United Kingdom

**Keywords:** Cyclodialysis cleft, Direct gonioscope, Hypotony, Ocular trauma.

## Abstract

**Aim:**

We present a novel surgical technique for repair of persistent and symptomatic cyclodialysis clefts refractory to conservative or minimally invasive treatment.

**Background:**

Numerous surgical techniques have been described to close cyclodialysis clefts. The current standard approach involves intraocular repair of cyclodialysis clefts underneath a full-thickness scleral flap.

**Technique:**

Our technique employs intraoperative use of a direct gonioscope to guide a nonpenetrating surgical repair. Subsequently, a significantly less invasive, nonpenetrating technique utilizing a partial-thickness scleral flap can be performed that reduces potential risks associated with intraocular surgery. The direct gonioscope is also used for confirmation of adequate surgical closure of the cyclodialysis cleft prior to completion of surgery. This technique has been successfully carried out to repair traumatic chronic cyclodialysis clefts associated with hypotony in two patients. There were no significant adverse events as a result of using this technique.

**Conclusion:**

The novel technique described increases the likelihood of successful and permanent repair of cyclodialysis clefts with resolution of symptoms associated with hypotony, through direct intraoperative visualization of the cleft.

**Clinical significance:**

Gonioscopically guided nonpenetrating cyclodialysis cleft repair offers significant benefits over previously described techniques. Advantages of our technique include gonioscopic cleft visualization, enabling accurate localization of the area requiring repair, and subsequent confirmation of adequate closure of the cleft. Using a partial-thickness scleral flap is also less invasive and reduces risks associated with treatment of this potentially challenging complication of ocular trauma.

**How to cite this article:**

Rodrigues IAS, Shah B, Goyal S, Lim S. Gonioscopically Guided Nonpenetrating Cyclodialysis Cleft Repair: A Novel Surgical Technique. J Curr Glaucoma Pract 2017;11(1):31-34.

## INTRODUCTION

A cyclodialysis cleft, first described by Fuchs^1^ in 1900, is an uncommon finding where there is separation of the longitudinal ciliary muscles of the ciliary body from the sclera spur. It often occurs secondary to ocular trauma^2^ and frequently results in symptomatic hypotony from the direct channel, i.e., formed, which allows unrestricted uveoscleral flow into the suprachoroidal space.^3^

Cyclodialysis clefts mostly recover spontaneously (especially if they involve less than 90° of the ciliary body) or with conservative management, such as prolonged treatment with 1% atropine sulfate twice a day.^4^ However, if the hypotony persists despite these measures, interventions, such as laser photocoagulation,^5,6^ trans-scleral diathermy,^7,8^ or cryotherapy^9^ can be attempted to close the cleft, with surgery reserved for cases that remain refractory.

The first surgical technique for repairing a traumatic cyclodialysis cleft was reported in 1952 by Vannas and Bjorkenheim.^10^ Numerous different techniques have been described for surgical closure of a cyclodialysis cleft in order to normalize the intraocular pressure (IOP). These all follow a similar principle of eliminating the cyclodialysis space by trying to restore apposition of the detached ciliary body back to the scleral spur. The most common surgical approach of direct cyclopexy involves raising a partial-thickness scleral flap over the cyclodi-alysis cleft. The cleft is directly visualized by creating a full-thickness incision under the partial-thickness scleral flap. The ciliary body can then be sutured to the scleral spur to close the cleft and the partial-thickness scleral flap is finally sutured.^3,11^

Direct cyclopexy involves rather invasive surgery with significant potential associated risks, such as supracho-roidal hemorrhage and endophthalmitis. Although the surgical approach of direct cyclopexy has high success rates,^3^ there, remains scope for further improvement and modification of the surgical technique. We describe a novel technique where intraoperative visualization of the cyclodialysis cleft with a direct gonioscope is combined with a nonpenetrating repair.

## TECHNIQUE

**Figs 1A to C:**
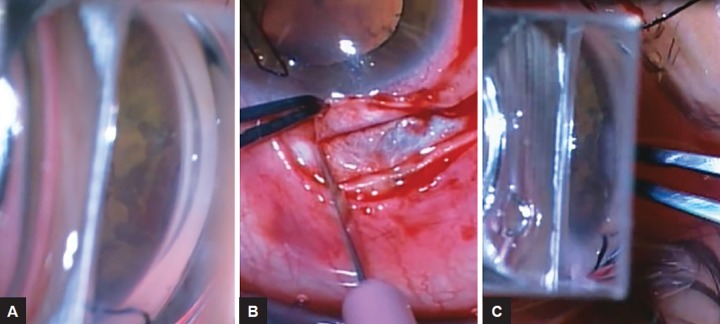
(A) Intraoperative view with direct gonioscope of cyclodialysis cleft (black arrow) with scars from previous thermal laser visible on the iris; (B) partial-thickness scleral flap is created overlying the cyclodialysis cleft; and (C) center of the cyclodialysis cleft is identified using a direct gonioscope with indentation of the sclera (white arrow) and marked with pen

The procedure may be carried out under local anesthesia with a subtenon block under usual aseptic surgical conditions. Topical pilocarpine 2% is instilled to obtain the best view of the angle under pharmacologically induced meiosis. The anterior chamber is also inflated with a viscoelastic to ensure that the full extent of the cleft is visualized gonioscopically, as accurate examination of cyclodialysis clefts may be difficult in a hypotonous eye. A direct gonioscope, in this case the Mori upright surgical gonio lens (Ocular Instruments), can then be used to identify the location of the cleft and fully recheck the rest of the angle to ensure that there are no other abnormalities (Fig. 1A).

A corneal traction suture is inserted followed by conjunctival dissection overlying the cyclodialysis cleft. A 50% thickness limbal-based scleral flap is then created over the cleft (Fig. 1B). The exact location of the center of the cleft should be identified using the direct gonioscope combined with indentation of the sclera (Fig. 1C) and then marked with a pen.

**Figs 2A to C:**
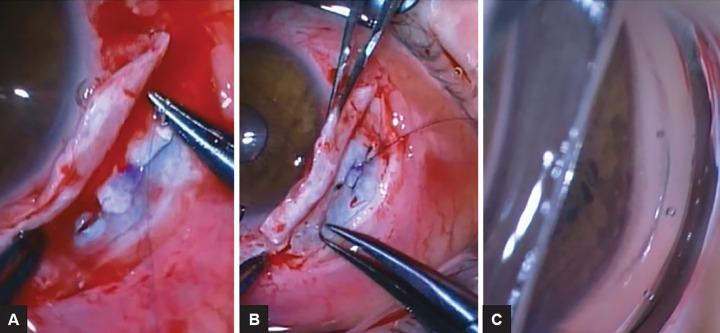
(A) Full-thickness 8/0 nylon suture is inserted through the sclera parallel to the limbus across the marked central point of the cyclodialysis cleft; (B) additional full-thickness 8/0 nylon sutures are added parallel to the limbus in order to fully close the cyclodialysis cleft; and (C) complete closure of the cyclodialysis cleft is confirmed intraoperatively with a direct gonioscope prior to closure of the scleral flap and conjunctiva

A full-thickness 8/0 nylon suture is then inserted through the sclera parallel to the limbus, traversing the marked central point of the cyclodialysis cleft (Fig. 2A). Closure of the cleft is checked with the direct gonioscope and additional full-thickness 8/0 nylon sutures are added parallel to the limbus, if required (Fig. 2B). Closure of the cyclodialysis cleft should then be confirmed with the direct gonioscope (Fig. 2C) before closure of the partial-thickness scleral flap and conjunctiva with 10/0 nylon sutures.

Postoperative treatment involves frequent topical steroids, such as guttae dexamethasone 0.1% six times per day and oral acetazolamide 250 mg twice daily in anticipation of high IOP in the immediate postoperative period due to any residual viscoelastic-reducing aqueous outflow. The topical steroid and acetazolamide can then be slowly tapered over 6 weeks after the surgery according to clinical response.

This surgical technique has been used in two patients to date. The first was a 42-year-old male who had blunt trauma to the right eye following a fall 18 months previously. The right visual acuity was reduced to 6/24 (20/80). The left eye had no abnormalities and a visual acuity of 6/5 (20/16). The right anterior chamber was shallow with the Van Herick anterior chamber depth measured at 10% (compared with the 50% in the left eye), with 360° closed angle on indirect slit lamp gonioscopy, but no peripheral anterior synechiae. The IOP was 4 and 13 in the right and left eyes respectively, with hypotony maculopathy present in the right eye.

Topical guttae atropine 1% b.d. did not result in any improvement, and laser photocoagulation was, therefore, attempted, but without any successful closure of the cleft. He then underwent surgical repair of the cyclodialysis cleft using the gonioscopically guided, nonpenetrating technique described earlier.

On the first postoperative day, the visual acuity was 3/60 (20/400) with corneal folds and poor view of the fundus, and the IOP was 6 mm Hg. However, 6 weeks postoperatively, the vision had improved to 6/12 (20/40); the IOP was 10 mm Hg, with the cleft remaining close on indirect slit-lamp gonioscopy.

The second patient also had blunt injury to the right eye; in this case, it was caused by a direct impact on the orbit with a golf ball. This resulted in hypotony, with an IOP of 4 mm Hg. The anterior chamber was shallow and there was 360° of appositional angle closure on indirect slit lamp gonioscopy. There was associated maculopa-thy and optic disk edema, with vision reduced to 6/36 (20/120).

This persisted for 6 months and was refractory to topical treatment with guttae atropine 1% b.d., so surgical repair of the cleft was carried out using the technique that we have described. On the first day postoperatively, the IOP was raised at 62 mm Hg, but this was reduced with topical IOP-lowering medications. Two weeks after surgery, the IOP was 18 mm Hg without requiring any IOP-lowering medication. There was a gradual reduction in the macular edema and optic disk edema, and 10 months after surgery, there was complete resolution. Visual acuity had improved to 6/9 (20/30) and the IOP was stable at 14 mm Hg.

## DISCUSSION

Gonioscopy in cyclodialysis clefts has traditionally only been used in the outpatient setting to visualize the drainage angle when diagnosing and then monitoring the cleft. However, a direct gonioscope, such as the Mori upright surgical gonio lens, the Koeppe diagnostic gonio lens, or the Swan Jacob gonioprism can be utilized intraopera-tively to accurately identify the location of the cyclodialysis cleft and guide placement of sutures. A direct gonioscope can also be used after suturing of the cleft to confirm that the cleft has been adequately closed. It also enables the precise effects of adjustment of sutures or placement of additional sutures to be immediately visualized.

Previously described techniques to repair cyclodialy-sis clefts have suggested full-thickness incision through the sclera in order to directly visualize the ciliary body prior to suturing it to the scleral spur. Indeed, the uncertainty of the precise location of the cleft means that a larger incision has been recommended that overlaps each side of the estimated end of the cleft by up to 2 mm.^12^ However, use of a direct gonioscope to visualize the cyclodialysis cleft enables the employment of a nonpenetrating technique. This offers significant advantages in safety as intraocular surgery is avoided, with associated reduction in the risk of serious complications, such as suprachoroi-dal hemorrhage and endophthalmitis. The less invasive surgery is likely to require less time in theater and also lead to increased speed of the postoperative recovery.

When undertaking any procedure to attempt closure of a cyclodialysis cleft, it is important to have close follow-up in the postprocedure period. It has been well documented that the IOP is unpredictable and has a tendency to be very high in the first week following the procedure, if there is no vigilant medical management of IOP.^13,14^ This IOP “spike” is in fact suggestive that the cleft has been successfully closed. This is because the aqueous humor that was previously bypassing the normal drainage pathway through the cyclodialysis cleft is now required to drain through the trabecular meshwork, which had been downregulated. In cases of cyclodialysis cleft following ocular trauma, it is also important to know that significant damage to the drainage angle is likely to have occurred.^15^ Therefore, long-term regular monitoring of IOP is necessary.

Although there are clear potential advantages of this technique and evidence that it can be successfully implemented in clinical practice, there are some potential limitations. Most importantly, as the technique is so critically dependent upon good visualization of the cyclodialysis cleft internally, it would not be suitable for patients who do not have a clear cornea or where the angle is obscured, e.g., by a persistent hyphema or by a shallow anterior chamber that cannot be adequately deepened with injection of a viscoelastic into it. This technique does require a blind pass through the ciliary body, which may pose potential problems during the repair of larger clefts, as there is risk of hemorrhage or damage to the adjacent structure. Finally, further repetition of this technique in a greater number of patients should be evaluated to provide true long-term efficacy and safety.

## CONCLUSION

In our experience, a gonioscopically guided nonpenetrating technique can be safely and successfully undertaken to repair chronic symptomatic cyclodialysis clefts that are refractory to medical and other noninvasive therapies. Careful management is necessary in the postoperative hypertensive phase, but IOP can be permanently normalized with full resolution of symptoms offering a good long-term visual prognosis.

## CLINICAL SIGNIFICANCE

The novel gonioscopically guided nonpenetrating cyclo-dialysis cleft repair technique that we have described and successfully performed offers two significant advances over other approaches that have previously been proposed. Firstly, the precision of gonioscopically guided visualization and secondly, the safety of a nonpenetrating technique. These are both important in ensuring success of the surgery as the rarity of cyclodialysis clefts requiring surgical repair, coupled with the technically demanding surgery, means that a more simple and safe technique than the current standard is a significant innovation.

## References

[1] Fuchs E (1900). Ablosung der Aderhaut nach Staaroperation.. Graefes Arch Clin Exp Ophthalmol.

[2] Malandrini A, Balestrazzi A, Martone G, Tosi GM, Caporossi A (2008). Diagnosis and management of traumatic cyclodialysis cleft.. J Cataract Refract Surg.

[3] Kiichle M, Naumann GO (1995). Direct cyclopexy for traumatic cyclodialysis with persisting hypotony: report in 29 consecutive patients.. Ophthalmology.

[4] Ormerod LD, Baerveldt G, Sunalp MA, Riekhof FT (1991). Management of the hypotonous cyclodialysis cleft.. Ophthalmology.

[5] Joondeph HC (1980). Management of postoperative and posttraumatic cyclodialysis clefts with argon laser photocoagulation.. Ophthalmic Surg.

[6] Harbin TS Jr (1982). Treatment of cyclodialysis clefts with argon laser photocoagulation.. Ophthalmology.

[7] Barasch K, Galin MA, Baras I (1969). Postcyclodialysis hypotony.. Am J Ophthalmol.

[8] Maumenee AE, Stark WJ (1971). Management of persistent hypotony after planned or inadvertent cyclodialysis.. Am J Ophthalmol.

[9] Krohn J (1997). Cryotherapy in the treatment of cyclodialysis cleft induced hypotony.. Acta Ophthalmol Scand.

[10] Vannas M, Bjorkenheim B (1952). On hypotony following cyclo-dialysis and its treatment.. Acta Ophthalmol (Copenh).

[11] Kato T, Hayasaka S, Nagaki Y, Matsumoto M (1999). Management of traumatic cyclodialysis cleft associated with ocular hypotony.. Ophthalmic Surg Lasers.

[12] Parikh RS, Parikh SR, Thomas R (2008). Surgical repair of cyclodialysis cleft.. J Curr Glaucoma Pract.

[13] Agrawal P, Shah P (2013). Long-term outcomes following the surgical repair of traumatic cyclodialysis clefts.. Eye.

[14] Ormerod LD, Baerveldt G, Green RL, Weinstein GW (1986). Cyclodialysis clefts: natural history, assessment and management.. Open angle glaucoma..

[15] Brooks AM, Troski M, Gillies WE (1996). Noninvasive closure of a persistent cyclodialysis cleft.. Ophthalmology.

